# Varying Opinions on Who Deserves Collectively Financed Health Care Services: A Discrete Choice Experiment on Allocation Preferences of the General Public

**DOI:** 10.1177/0046958017751981

**Published:** 2018-02-14

**Authors:** Maartje J. van der Aa, Aggie T. G. Paulus, Mickaël J. C. Hiligsmann, Johannes A. M. Maarse, Silvia M. A. A. Evers

**Affiliations:** 1 Maastricht University, The Netherlands

**Keywords:** health insurance, resource allocation, public opinion, choice behavior, surveys and questionnaires, experimental design, discrete choice experiment, The Netherlands

## Abstract

In Europe, health insurance arrangements are under reform. These arrangements redistribute collectively financed resources to ensure access to health care for all. Allocation of health services is historically based on medical needs, but use of other criteria, such as lifestyle, is debated upon. Does the general public also have preferences for conditional allocation? This depends on their opinions regarding deservingness. The aim of this study was to gain insight in those opinions, specifically by examining the perceived weight of different criteria in allocation decisions. Based on literature and expert interviews, we included 5 criteria in a discrete choice experiment: need, financial capacity, lifestyle, cooperation with treatment, and package/premium choice. A representative sample of the Dutch population was invited to participate (n = 10 760). A total of 774 people accessed the questionnaire (7.2%), of whom 375 completed it (48.4%). Medical need was overall the most important criterion in determining deservingness (range β = 1.60). Perceived deservingness decreased if claimants had higher financial capacity (1.26) and unhealthier lifestyle (1.04), if their cooperation was less optimal (1.05), or if they had opted for less insurance coverage (0.56). However, preferences vary among respondents, in relation to demographic and ideological factors.

## Introduction

All over Europe, both the scope^1^ and expenditures of social health insurance (SHI) have increased.^2^ Governments increasingly consider the expenditure trend to be unaffordable over time. To curb this trend, they discuss and initiate reforms of SHI. These reforms may put pressure on solidarity, which is one of the core values of SHI. Solidarity in SHI is the shared responsibility for financial risks of health care use of all individuals.^3^ Shared responsibility manifests itself in health care by collecting financial resources from all citizens and redistributing them to certain “agreed-upon individuals.”^4^ Without such solidaristic arrangements, health care services may not be accessible for all.^5^

There has always been debate about who are the “agreed-upon individuals” whom we want to be solidaristic with or, in other words, who is granted access to collectively financed health care resources. In the Netherlands, as in most Organisation for Economic Co-operation and Development (OECD) countries, access is historically based on the allocation criterion “medical need.”^6^ However, other allocation criteria, for instance, lifestyle, are currently the topic of the political and social debate.^7^ Adjustments in allocation criteria may affect access, subsequently the redistributive effect of SHI and eventually solidarity. Does the general public also have preferences regarding allocation which takes nonmedical criteria into account? This depends on their opinion about allocation in publicly financed social arrangements, which is highly influenced by their perceptions of deservingness.^8^

Deservingness is a concept that refers to moral judgments on who are the “agreed-upon individuals” whom we want to be solidaristic with. The central question in determining this group, and thus the question at the heart of social arrangements, is “who deserves to be allocated collectively financed health care services and why?”^9^ Deservingness of claimants depends on the specific situation of these claimants. Social policy research has shown that ill individuals, especially when older, are generally considered most deserving.^10^ In line with this deservingness opinion (subjective), health care allocation (objective) has always been primarily need-based. At the same time, several studies in the field of health economics identify allocation criteria beyond need.^11-13^ Over the years, social policy researchers have developed a comprehensive set of 5 criteria—characteristics of claimants—that are considered to determine the perceived deservingness of claimants.^9^ Beyond claimants’ necessity for support (need), people are considered deserving when they are “one of us” (identity) and when they have given or will give society something in return (reciprocity). Moreover, it is considered important that claimants try to control their need (control), and are docile and grateful when receiving support (attitude). Despite the extensive body of knowledge on deservingness in social policies, deservingness is an uncharted field in health care. Health care research has mostly focused on the efficiency part in the efficiency-fairness trade-off or investigated the influence of a single indicator on allocation preferences, for example, age. Therefore, this study addresses the following research question: Does variation in the values of deservingness criteria influence public opinion about deservingness for collectively financed health care services, and if so, how and how much?

Social policy research has shown that people with different demographic and ideological backgrounds place different emphasis on each of the deservingness criteria.^14^ Hence, deservingness of claimants is influenced not only by the claimant’s characteristics but also by the appraiser’s characteristics. Nevertheless, evidence is unequivocal about how the appraisers’ characteristics relate to deservingness opinions. For instance, levels of income and/or education—typically treated as measures of self-interest—have been related both positively and negatively to welfare support. On one hand, an inverse relationship between income level and welfare state support is explained by the theory that individuals with lower income become more dependent on the system and will therefore support it.^15^ On the other hand, individuals are theorized to be also supportive toward the welfare state due to their experience that it has aided them in reaching their position.^16^ Moreover, in the case of health care, there has also been argued that income level is not related to welfare support, because illness is distributed randomly as a result of which everyone has the risk to become dependent.^8^ Regarding the influence of ideological background of appraisers on their deservingness opinions, literature is less ambiguous. Respondents’ political stance has been found to influence deservingness opinions in different social policies.^17,18^ However, it is unknown whether deservingness opinions in health care also vary among individuals. Therefore, it is unknown whether deservingness opinions in health care also vary among individuals. Therefore, this study additionally addresses the research question: What are the differences in health care deservingness opinions among subgroups with different demographic (eg, gender) and ideological factors (eg, political opinion)?

The previously described background shows that the body of knowledge on deservingness opinions stems mostly from social policy research. We will use that knowledge—for instance, the 5 deservingness criteria—as a starting point to investigate deservingness opinions in the field of health care, about which not much is known. Deservingness underlies health care allocation policies that are currently under reform. To inform such decisions, this study aims to gain insight in deservingness opinions of the general public regarding health care, meaning the role of different criteria in allocation.

## Method

This study conducts a discrete choice experiment (DCE) to retrieve preferences for these criteria. A DCE is a method that is able to elicit group-level preferences and to quantify trade-offs between preference criteria.^19,20^ We conducted the experiment in the Netherlands.

### Discrete Choice Experiments

The technique of a DCE is based on the premise that welfare claimants can be described by a number of characteristics (ie, attributes) and that their deservingness is influenced by the variations (ie, levels) within these attributes.^21^ Specific combinations of attribute levels are lined-up side-by-side, and respondents are asked to state which of the alternatives they find most deserving. These choices require trading-off among attributes. Statistical analysis makes these trade-offs explicit by retrieving the weight different attributes have in these choices.

Although originating in the field of economics, DCEs are increasingly used in health care, with a wide range of applications.^22^ In conducting our experiment, we followed renowned DCE guidelines that have been developed for use in health care research.^20,23^ The experiment consists of 4 steps: (1) identification and selection of attributes and levels, (2) design, (3) data collection, and (4) data analysis.

### Attributes and Levels

Identifying and selecting attributes and levels is an important step to guarantee the reliability of a DCE. We reviewed relevant literature by searching the terms “deservingness criteria” in EBSCOhost and Google Scholar. It showed that the concept of deservingness has much developed in the last 3 decades, mainly in the field of social policies (eg, by De Swaan^24^). Currently, the 5 criteria of Van Oorschot,^9^ also mentioned in the “Introduction” section, are widely adopted. However, these criteria of need, identity, control, attitude, and reciprocity are not developed for the field of health care. Therefore, these criteria were used as a starting point and discussed in expert interviews (n = 12) to critically assess their applicability to health care. This exercise showed that deservingness criteria require more nuance in health care than in the loss-of-income insurance arrangements they originate from. To make them applicable to health care, we made 5 adjustments to the criteria of Van Oorschot.

First, the need criterion was disentangled into a medical and financial component, because the medical component is insufficiently reflected by the general need criterion. Second, identity was excluded in this study, because identity-related allocation is outlawed based on discrimination legislation. Third, the criterion “control” was subdivided into lifestyle (behavior prior to the onset of an illness) and cooperation (behavior during treatment), because this was considered a relevant distinction in health care. Fourth, the criterion “attitude” was excluded because it is impracticable for use in future policies—which was a requirement for inclusion—because it is hard to operationalize attitudes. Finally, reciprocity was conceptualized according to the quid pro quo principle, which refers in health insurance to members’ contributions to social insurance and their relation to allocation. In summary, based on literature and expert interviews, we selected 5 attributes for the experiment, which is a feasible number of attributes to conduct a DCE: need, financial capacity, lifestyle, cooperation with treatment directions, and choice of package/premium (Table 1).

**Table 1.** Deservingness Criteria Used in the Experiment.

Attributes	Levels	Coefficient in analyses
Medical need (severity of illness) The impact that an illness has on the quality of life in event of nontreatment	Low (20% loss in quality of life)	β_1_
	Average (40% loss in quality of life)	
	High (60% loss in quality of life)	
Financial capacity Financial resources available to cope with health care expenses	Low	Reference level
	Moderate	β_2_
	High	β_3_
Lifestyle The patient’s behavior prior to the onset of illness	Optimal	Reference level
	Suboptimal	β_4_
Cooperation The patient’s behavior during treatment	Optimal	Reference level
	Suboptimal	β_5_
Choice of package/premium The chosen level of coverage (and accordingly premium) of the health insurance policy	High	Reference level
	Medium	β_6_
	Low	β_7_

We also used literature as a starting point for level selection and additionally consulted methodological experts (n = 5), which resulted in the selection of 2 or 3 levels per attribute. The criterion of “medical need” is commonly expressed by levels that represent a specific disease. However, we phrased it into more abstract terms—severity in terms of loss in quality-adjusted life years (QALYs)—because labels of specific diseases could wake perceptions/images of these diseases instead of actual opinions about the deservingness criterion. The levels of lifestyle and cooperation were not phrased as “optimal” and “obstructing,” because the latter was considered both unlikely in practice and likely to disrupt the results by the dominant view of being undeserving in case of obstruction treatment. Instead, we used “suboptimal” because it is more realistic.

### Designing Choice Sets and Questionnaire

**Figure 1.**
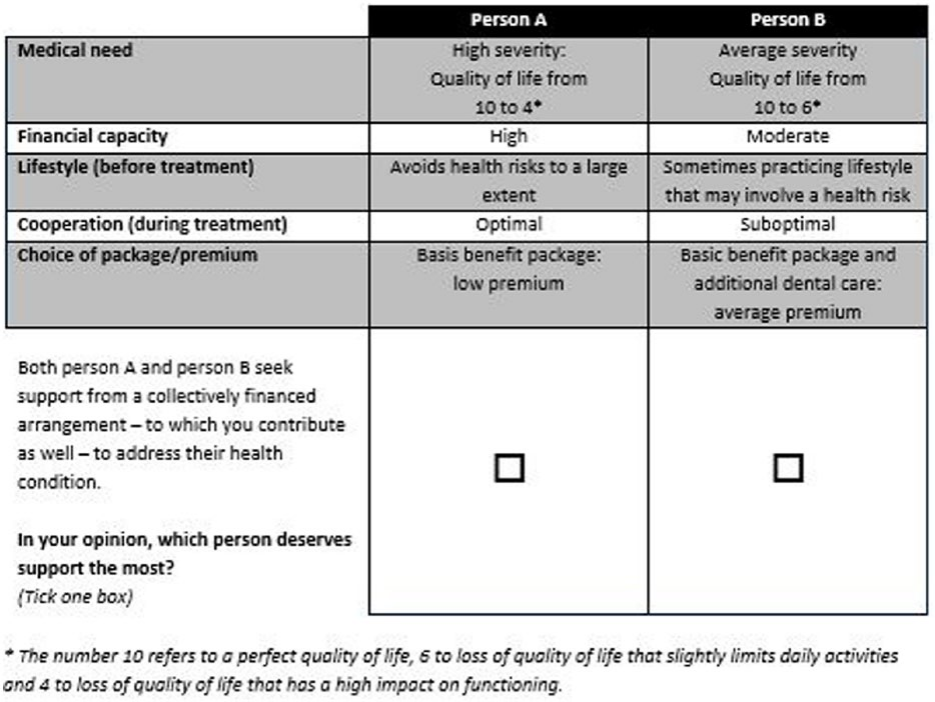
Example choice set in the discrete choice experiment.

Out of the selected attributes and levels, 11 664 unique alternatives (3^2^× 3^2^× 3^2^ × 23 × 23) and numerous choice sets—each consisting of 2 alternatives—could be constructed, which could not all be presented to the respondents. A Bayesian efficient experimental design was used to select a feasible number of 9 choice sets. D-efficiency was maximized in this design, which is in line with the DCE guidelines mentioned previously. We used Ngene software (version 1.1.1) to do so. In each choice set, the respondent has to identify the person who is most deserving of 2 hypothetical persons (alternatives) who differ according to the attributes. Figure 1 shows an example choice set of the DCE.

The final questionnaire contained 11 choice sets, which were the same for all respondents. Nine choice sets were part of the experiment. In addition, the questionnaire included 2 validity tasks. First, we included a dominance test, which presented the most deserving scenario (highest need, optimal cooperation, etc) and the least deserving scenario. Second, we ensured test-retest reliability by presenting one of the choice sets again. All choice sets were presented as unlabeled choices between person A and person B, which encourages respondents to state their preference by only trading-off attribute levels.^25^ Respondents could not opt out in these questionnaire items—forcing them to make a choice—to make the experiment realistic: Policy makers also have to make these allocation decisions, because resources can be allocated only once.

In addition, the questionnaire contained several questions to obtain information on respondents’ personal characteristics and welfare attitude in general. The questionnaire was developed in Dutch and included a comprehensive explanation of the attributes and levels at the beginning, also providing concrete examples to make clear what each abstract level meant. It was designed in Qualtrics online survey software (version 7812362). The questionnaire was piloted (n = 5) to check interpretation, face validity, and layout. Only minor changes were made in phrasing and layout.

### Data Collection and Respondents

A representative panel sample of the Dutch population (sex, age, region, and educational level) was invited to participate in the experiment. To prevent selective response among the invitees, batches of samples were drawn that corrected for overrepresentation in the sample at that point. The samples were drawn by CG Selecties based on the gold standard developed by the Organization for Market Research (MOA) in collaboration with Statistics Netherlands (CBS). Potential respondents were approached in batches, which allowed for adjusted targeting and thus overcoming participation bias. A total of 10 760 members of the panel received an invitation through the Qualtrics email function. Their data were collected by the same online survey software in July 2015.

### Statistics and Data Analysis

Choice data were analyzed statistically based on random utility theory, which assumes that respondents made rational decisions, that is, maximizing utility.^26^ Utility, a latent trait describing deservingness, can be decomposed into a constant, attribute levels that each has a preference coefficient, and an error term.^20^ Preferences of respondents (*i*) are statistically represented by utility (*U*), which is the sum of their preference scores for attributes/levels expressed in their choices (*j*) in different choice sets. The term η is the error term capturing unexplained variation between respondents. We used a panel model to control for repeated observations within the same individual. This led to the following utility function:

$$\begin{array}{l} U_{ij}\;\;\;=Constant\;+\\ \left(\beta_{1}+\eta_{1i}\right)\;\cdot \;Need\;\cdot \;\;j\;+\\ \left(\beta_{2}+\eta_{2i}\right)\;\cdot \;FinancialCapacity_{Moderate}\cdot \;\;j\;+\;\left(\beta_{3}+\eta_{3i}\right)\cdot \;FinancialCapacity_{High}\cdot \;\;j\;+\\ \left(\beta_{4}+\eta_{4i}\right)\;\cdot \;Lifestyle_{Suboptimal}\cdot \;\;j\;+\\ \left(\beta_{5}+\eta_{5i}\right)\;\cdot \;Cooperation_{Suboptimal}\cdot \;\;j\;+\\ \left(\beta_{6}+\eta_{6i}\right)\cdot \;PackagePremium_{Medium}\cdot \;\;j\;+\;\left(\beta_{7}+\eta_{7i}\right)\;\cdot \;Package_{PremiumLow}\cdot \;\;j. \end{array}$$

Preference or utility for a certain choice alternative can therefore be defined as a sum of preference scores for attributes/levels within this alternative. Dummy coding was used for all attributes, except for “need.” The first level of each of the dummy coded attributes is the reference level, which means that they are left out of the function. The attribute need was operationalized as a continuous variable (percentage of loss in quality of life), even though choice sets contained only 3 alternatives (60%, 40%, or 20%). Such a specification of numerical attribute levels into a continuous variable has been explained by Hauber et al.^27^

Betas in the utility function represent the weight given to the respective attribute. The weight is relative, and should thus be interpreted in relation to weights of other attributes within the same model. A higher beta parameter indicates that the respective attribute has a higher weight in determining which of the alternatives was considered more deserving. We used a mixed logit model (1000 Halton draws) to determine the beta parameters and other components in the utility function. This model assumes that parameters are randomly distributed. A mixed logit model therefore allows assessment of preference heterogeneity by estimating the standard deviation of each beta’s distribution.

Relative importance of attributes can also be expressed by the proportion that an attribute’s variation has in explaining the variation in utility,^28^ which is a measure easier to interpret, facilitating comparison between weights of different models. Relative importance is derived by dividing the range of betas of an attribute’s levels by the sum of the ranges of all attributes’ levels within the model.

Only complete responses of respondents that passed the dominance test were included in these statistical analyses, which were performed in Nlogit econometric software (version 5).

### Subgroup Analysis

Several subgroup analyses were conducted to assess the impact of covariates on preferences, in particular the influence of demographic and ideological variables, which have been associated with deservingness opinions in other social policies. For instance, respondents aged between 46 and 64 preferred more conditional allocation preferences in comparison with younger and older respondents, while respondents of lower socioeconomic status and/or a history of receiving benefit have shown a preference for less conditional allocation, mainly regarding the control attribute.^17^ The same experiment found that those on the political right prefer conditional allocation based on reciprocity, which is represented by our criterion of premium/package choice. We conducted subgroup analysis on variables that were identified to influence deservingness—in literature—and that were available in the dataset: age, gender, education, income, opinion about the state’s responsibility for the health system, and political preference on the left-right continuum.

To analyze subgroup variation, we created dummy variables for the respondents’ characteristics that potentially could influence deservingness opinions. We estimated joint models according to the utility formula, in which each included the dummy variable (one per model) and its interaction coefficient in each term. These models assessed whether coefficients of the attribute levels varied among the subgroups. Dummy coding on the variables age, education, income, and perceived state responsibility for health care was done by a subdivision that approximated an equal number of respondents in each group. The positioning of political parties in the political landscape by experts in the recent study of Otjes^29^ provided the basis for the Dutch left-right spectrum used in this article. The labor party (PvdA), socialist party (SP), green party (GL), Christian socialists (CU), and the single-issue animal rights party (PvdD) were positioned left, whereas the Christian democrats (CDA), protestant orthodox (SGP), left-wing liberals (D66), right-wing liberals (VVD), and right-wing populists (PVV) were positioned right. The researchers placed the new pensioners’ party (50PLUS) on the left due to their socialist political stance on demographic topics. The opt-out, nonresponse, and the options “rather not tell” and “other” were not dummy coded.

## Results

The response rate was 7.2%, which means that 774 members participated in the study. A total of 375 respondents completed all choice tasks (48.4%). Out of the incomplete responses, only 5 respondents filled out at least half of the choice tasks, which is needed to get reliable results. Sensitivity analysis showed that inclusion of these 5 incomplete cases did not affect the results. Within the 375 complete responses, there were 30 respondents who did not pass the dominance test. These responses were excluded for analysis, upon which data of 345 cases were analyzed. The respondents covered a wide variety of population groups by age, educational levels, and political stands (Table 2). The characteristics of the respondents are similar to those of the Dutch population, although there was some oversampling of tertiary educated individuals. In general, the respondents considered the welfare state highly responsible for health care (on a scale from 1 to 10: μ = 7.95; σ = 1.687).

**Table 2.** Baseline Characteristics of Respondents.

	Data set	Dutch population^a^
n	345	16 900 726
Questionnaire duration (in mm:ss) (5% trimmed mean)	16:42	—
Gender		(2015)
Male	161 (46.7%)	8 372 858 (49.5%)
Female	184 (53.3%)	8 527 868 (50.5%)
Age, y, mean	46.4 (±14.9)	41.3
Educational level		(2012)
Primary (basisschool)	5 (1.4%)	890 (8.2%)
Lower secondary or equivalent (VMBO/LBO)	49 (14.2%)	2 453 (22.5%)
Upper secondary or equivalent (MBO/HAVO/VWO)	148 (42.9%)	4 432 (40.7%)
Tertiary (HBO/WO)	143 (41.4%)	3 109 (28.6%)
Income level (net)		(2014)^b^
No income or <€750 per month	31 (8.9%)	2 561 000 (19.8%)
€750-€1500 per month	102 (29 .6%)	3 036 000 (23.5%)
€1500-€3000 per month	136 (39.4%)	4 241 000 (32.8%)
€3000-€5000 per month	26 (7.5%)	1 212 000 (9.4%)
>€5000 per month	2 (0.6%)	1 864 000 (14.4%)
Opt-out	48 (13.9%)	—
Health care use (last year)		
No	115 (33.3%)	—
Yes	230 (66.7%)	—
Health care use peers (last year)		
No	127 (36.8%)	—
Yes	218 (63.1%)	—
Government’s responsibility for health care		
(0 = no responsibility; 10 = full responsibility) (mean)	7.95 (±1.69)	—
Political opinion		(2012)
No preference	69 (20.0%)	25% (blank vote)
Labor Party (PvdA)	28 (8.1%)	19%
Socialist Party (SP)	52 (15.1%)	7%
Left-wing Liberals (D66)	47 (13.7%)	6%
Right-wing Liberals (VVD)	43 (12.5%)	20%
Right-wing Populism (PVV)	33 (9.6%)	8%
Christian Democrats (CDA)	16 (4.6%)	6%
Other	38 (11.0%)	9%
Opt-out	19 (5.5%)	25% (no vote)

*Note.* Values represent crude numbers instead when it is stated that they represent means. In case of crude numbers, the values in brackets represent percentages. In case of means, values in brackets represent standard deviation.

a Data of Statistics Netherlands (CBS).

b Data were available per year and grouped by €10 000. The thresholds of the income groups in the experiment were multiplied by 12 and linked to the closest income group in the available data of the Dutch population.

### Trade-Offs Between Deservingness Criteria

Betas of the attributes determining the latent trait of health care deservingness (*U*) are reported in Table 3. They are derived from a mixed logit model. Betas represent the weight that each level has in influencing the deservingness trait, in comparison with the weight of other levels. The results show that the levels of medical need have the highest coefficients (range β_1_ = 1.60). The positive value of the beta per percentage indicates that higher levels of need are considered more deserving. A drop of 1% in need results in claimants being considered 0.04 less deserving of support, indicating a maximum utility/deservingness of 2.4. All other criteria have a negative sign, and thus, deservingness decreases. Financial capacity decreases deservingness the most (range 0-β_3_ = 1.26), but cooperation (0-β_5_ = 1.05) and lifestyle (0-β_4_ = 1.04) also influence deservingness. For instance, having high financial capacity decreases perceived deservingness by 0.77 in comparison with moderate financial capacity. A claimant’s choice of premium/package (β_7_ = 0.56) influences deservingness the least. If someone opts for a broader benefits scheme, he or she is considered 0.27 more deserving of receiving publicly financed support than someone who opts for a medium package. In addition, we calculated the relative importance of attributes, indicating the role of an attribute in deservingness decisions. These proportional measures show that need determines deservingness for about 30%, whereas the other attributes had a smaller role (10%-23%).

**Table 3.** Betas and Relative Importance of Health Care Deservingness Criteria.

Attribute	Level	Weight				Relative importance^a^ (%)
		Beta	SE	SD	Range betas	
Medical need (%)	20% loss	0.04*** (per % loss in QALY)	0.00	0.05***	1.60	30
	40% loss					
	60% loss					
Financial capacity (categorical)	Low	(ref)			1.26	23
	Moderate	−0.49***	0.09	0.01		
	High	−1.26***	0.11	0.77***		
Lifestyle (categorical)	Optimal	(ref)			1.04	19
	Suboptimal	−1.04***	0.08	0.60***		
Cooperation (categorical)	Optimal	(ref)			1.05	19
	Suboptimal	−1.05***	0.09	0.90***		
Premium/package choice (categorical)	High	(ref)			0.56	10
	Medium	−0.27***	0.08	0.03		
	Low	−0.56***	0.09	0.54***		

*Note.* QALY = quality-adjusted life years.

a Percentage total does not add up to 100% due to rounding.

*** *P* < .01.

**Figure 2.**
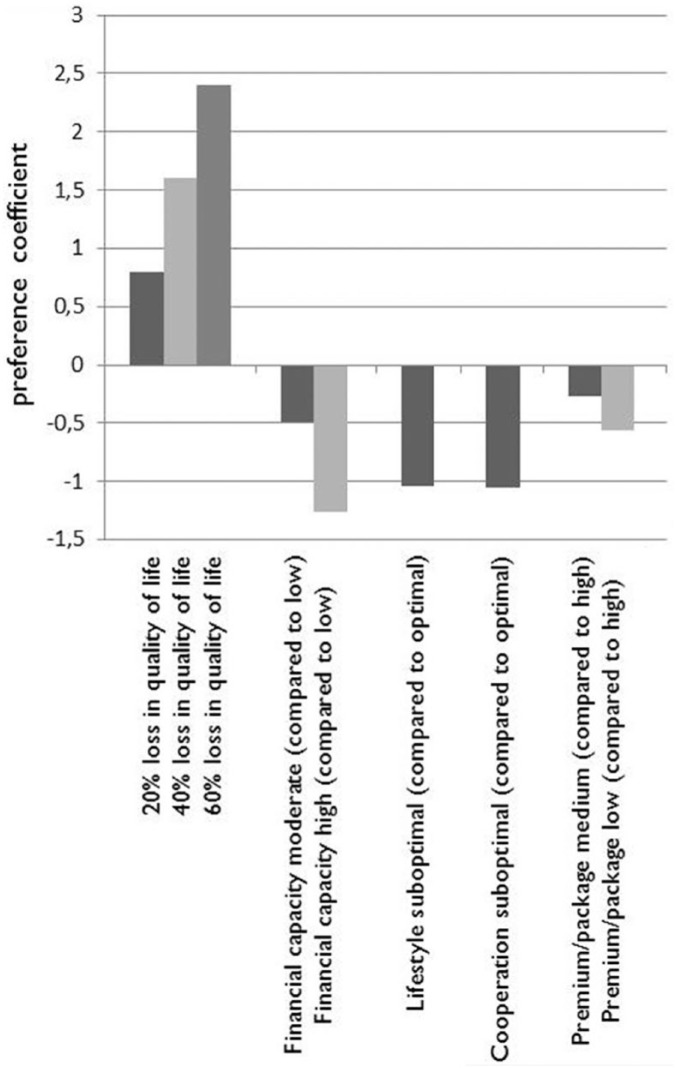
Visualized trade-offs of the health care deservingness criteria.

The betas of different levels also provide information about the trade-off of different criteria. The weight of medical need in *U* (β_1_) is 2.4 when an illness causes a 60% loss of quality of life, but 0.8 when that loss is 20% (see Figure 2), which shows the role of medical need in deservingness decisions depends on the exact level of need. Comparing the betas of medical need with those of the nonmedical criteria indicates that medical need is the most important criterion when the level of need is above approximately 32% loss of quality of life (corresponding with a β of 1.28), as the larger bars on the left side of Figure 2 show. However, summing up the weights of the nonmedical criteria (the negatively valued bars in Figure 2) shows that jointly these nonmedical criteria can outweigh the need criterion – in case of lower levels. These trade-offs can be derived from the visualization of the betas in Figure 2.

Additionally, the standard deviation of each of the beta parameters was estimated to assess heterogeneity. Standard deviations were significant for most of the attributes, indicating that different respondents hold different deservingness opinions. Subgroup analysis allows for more detailed information on this heterogeneity.

### Subgroup Analysis

The results of all subgroup analyses can be found in Table 4. Females, younger respondents (≤45 years) and respondents having a higher socioeconomic status had significantly more conditional views on health care allocation regarding the lifestyle (−0.36 and −0.25) and cooperation (−0.41 and −0.23) of a claimant (both *P* < .05). There were also differences in subgroups regarding education and income, variables used to measure self-interest. Better educated respondents thought that claimants who choose smaller insurance packages were much less deserving than did respondents with lower education (*P* < .05). Subgroups on income differed significantly on the weight they assigned to lifestyle: Suboptimal lifestyle was blamed much more by those on high income than by those on low income (*P* < .05). Finally, the ideological characteristics of respondents showed to affect the weight of financial capacity and need: Respondents who consider the state highly responsible for health care prefer more need-based allocation (+0.03; *P* < .01) and allocation to be less affected by the financial capacity of claimants (–0.56; *P* < .05). Respondents on the political left considered claimants practicing suboptimal lifestyle and opting smaller insurance packages less deserving for collectively financed resources, but not to the same extent as did respondents on the right side of the political spectrum. However, respondents’ political stance on the left-right continuum did not significantly affect deservingness opinions.

**Table 4.** Betas and Relative Importance of Health Care Deservingness Criteria by Subgroups.

Attribute	Level	Difference in beta’s by subgroup					
		Female (vs male)	Age ≤45 (vs >45)	Tertiary education (vs other/no)	Income ≥€1500 (vs <€1500)	Responsibility state for health care high (vs low)	Political right (vs left)
Medical need	20% loss	–0.01 (per % loss)	–0.00 (per % loss)	0.01* (per % loss)	0.01 (per % loss)	0.03*** (per % loss)	0.01 (per % loss)
	40% loss						
	60% loss						
Financial capacity	Low	(ref)	(ref)	(ref)	(ref)	(ref)	(ref)
	Moderate	−0.11	0.04	−0.32*	0.20	−0.36**	0.13
	High	−0.34	−0.21	−0.18	0.37	−0.56**	0.37
Lifestyle	Optimal	(ref)	(ref)	(ref)	(ref)	(ref)	(ref)
	Suboptimal	−0.36**	−0.25**	−0.27*	−0.42**	−0.15	−0.29*
Cooperation	Optimal	(ref)	(ref)	(ref)	(ref)	(ref)	(ref)
	Suboptimal	−0.41**	−0.23**	−0.14	−0.15	−0.31*	0.13
Premium/package choice	High	(ref)	(ref)	(ref)	(ref)	(ref)	(ref)
	Medium	0.22	0.08	0.00	0.37**	−0.15	−0.06
	Low	0.28*	−0.01	−0.43**	−0.28	0.20	−0.36*

* *P* < .10. **P < .05. ***P < .01.

## Discussion

The aim of this study was to gain insight into the general public’s opinions regarding the deservingness of health care. Therefore, we investigated the importance of several allocation criteria in determining who deserves collectively financed health care services. A DCE elicited relative weights of 5 deservingness criteria among a representative sample of the Dutch population. The results show that the claimant’s medical need, financial capacity, lifestyle, cooperation, and insurance package/premium choice all shape deservingness opinions, but that they are emphasized differently. Medical need is considered the most important criterion. However, lifestyle, cooperation, and financial capacity of the claimant were significant criteria in deservingness choices as well, especially in cases of less severe medical needs. Moreover, the experiment showed that health care deservingness opinions vary among subgroups. The interaction models showed that demographic factors—such as age, gender, education, and income—mainly influenced emphasis on lifestyle and cooperation, while ideological factors changed the emphasis on the criteria need and financial capacity.

### Interpretation Results

Allocation policies in the Netherlands are traditionally need-based.^6^ The experiment showed that deservingness opinions of the general public are in line with this practice because opinions were mostly determined by a claimant’s medical need. However, the experiment shows that perceptions of deservingness are not only need-based. The weight of nonmedical criteria in determining deservingness indicates that claimants with medical needs are not viewed as unreservedly deserving and are also held responsible individually.

The trend toward a greater role of individual responsibility in policies^30^ could explain the results. After all, the experiment is about allocation of collectively financed resources, which is based on the principle of shared responsibility (solidarity). The results therefore show that medical needs of individuals are not always considered to be a shared responsibility. In other words, the respondents of the DCE also hold claimants individually responsible for their risk of health care use, which fits in the trend of increased individual responsibility in health care policies. However, these results need to be nuanced, because they refer to trade-offs: Nonmedical criteria become relatively more important in allocation of health care resources—indicating a shift to individual responsibility—when the claimant’s medical need is below a certain level of severity (approximately 32%). Therefore, the experiment shows that the financial risk of health care use due to more severe illnesses remains a shared responsibility. This corresponds with the widespread support for solidarity in health care among the Dutch population.^31,32^ However, under the surface, these studies also found restrictions on solidarity. Further research should investigate how this threshold of severity of disease—below which other criteria become relatively more important—could be interpreted in practice.

Regarding the subgroup analyses, this experiment shows that deservingness opinions regarding health care are also influenced by demographic and ideological factors, as in other social policies.^14^ However, in comparison with deservingness studies in other social policies,^17^ we did not find that the political stance of respondents had a significant effect on their preferences for either unconditional or conditional allocation of health care. Moreover, heterogeneity of results complicates interpretation, because there is not such a thing as “the” Dutch opinion, not even within subgroups that we could analyze. A latent class model would be helpful to investigate meaningful subgroups in the future.

### Strengths and Limitations

To our knowledge, this study is one of the first studies investigating prioritization of allocation criteria in health care and their trade-off. Its novelty lies in the focus on moral judgment, that is, views on *who* qualifies for collectively financed services. Many studies have been conducted on health care allocation, but these focus mainly on concepts such as costs, outcome (QALY), and efficiency,^11^ that is, *what* services should be financed collectively. Moreover, if these studies pay attention to moral judgment, it often involves only a single criterion, for example, lifestyle, that is weighed against efficiency measures instead of viewing judgment as a result of a trade-off between several criteria of fairness. This is in line with the efficiency-fairness trade-off,^33^ which explains that more efficient policies may result in less fairness and vice versa. However, balancing efficiency and fairness looks only at fairness in relation to efficiency measures. We acknowledge that they are related and that, for instance, lifestyle could be seen as an aspect having influence on the efficiency of certain treatments—potentially affecting deservingness opinions indirectly—as well as on deservingness opinions directly. Nevertheless, focusing on fairness only contributes to the aim of this study, that is, to gain insight in pure or a priori moral judgments on health care allocation. The experimental design and the sample, which represents the Dutch population, are strengths of this study.

Our study could have some limitations. First, the experiment was limited by the number of attributes that could be included. Although the identification and selection of criteria was done in line with DCE guidelines, we were able to only include the most important deservingness criteria, which resulted in the exclusion of other criteria that are also relevant and may even be more relevant at this time. Most notably are the criteria of age and ethnicity, which are all related to the criterion identity—whether a claimant is perceived to be “one of us.” Although we were not able to include these criteria, we highly recommend further research on the trade-off of these criteria with the criteria we have used in our study. A second limitation is that the selection of attribute levels could influence the measure of relative importance. For instance, formulating the lowest category of need by 5% severity would result in much lower levels of deservingness. We did not use these extreme levels to prevent a dominant attribute, which does not reveal much of the trade-off in which we are interested. Nevertheless, a consequence of the relationship between level-design and outcomes is that the measure of relative importance of attributes provides only an indication of the attributes importance, considering the specific range of levels used in the study. A third limitation is that this study was cross-sectional, which does not reveal information on deservingness opinions over time.

Another factor that complicates interpretation of the results is the abstract phrasing of levels. For instance, cooperation and lifestyle were operationalized by the levels optimal and suboptimal, because experts indicated that obstruction was an unrealistic level and would trigger dominant responses. However, this leaves the question for the interpretation of the results: What is suboptimal cooperation or lifestyle? Similarly, levels within the attribute need were formulated abstractly as well—that is, high severity instead of mentioning a specific disease. The experts indicated that labels of specific diseases would trigger existing images of diseases, which are based on media and personal experience. However, not labeling alternatives leaves open the debate about the results’ implications: Which are those less severe diseases that the general public considers less deserving? The abstract level formulation of the variables need, cooperation, and lifestyle are therefore useful in studying deservingness opinions but are hard to interpret in practice.

Furthermore, the data were collected in July 2015, when a new social support act had been implemented on January 1 of the same year. This act potentially affected the allocation of long-term care resources, which was expected to be restricted. Keeping this context in mind, we think that respondents may have been influenced to show preference for the unconditional allocation that they might fear losing. However, we did not observe opinions that opposed conditional allocation altogether, on the other hand.

Finally, it is unknown whether public opinion in other countries would correspond with what was found for the Netherlands in this experiment. We would like to invite our colleagues in other countries to conduct similar studies on their home ground for comparison.

### Contribution

Literature has shown that the general public agrees that some form of reform has to be implemented, but that subgroups do not agree on the form of these reforms.^34^ Governments search for policy reforms to curb the health care expenditure trend, and this study contributes to the discussion on the design of SHI reform.

The use of public opinion would respond to the trend of deliberation and public engagement in policymaking, which may be beneficial for the successful implementation of policies. The results of this study indicate that, especially in the case of less severe illnesses, the general public is in favor of for conditional allocation. Although the preferences found do not suggest that the general public also wants these preferences to be translated into allocation policies, it does give an indication that using nonmedical criteria in allocation policies may be supported. Such a policy change would be in line with the increased emphasis on individual responsibility, which is already being seen in public health.^35^ However, the Dutch need-driven SHI system does currently not allow nonmedical conditions to be used in allocation of services in the basic benefit package: The financial risk of using these services is considered to be a shared responsibility. In addition, the use of nonmedical allocation criteria in health care contrasts with traditional need-based allocation.^36^ Nevertheless, this study feeds the debate on reforming SHI and on the balance between shared responsibility (solidarity) and individual responsibility for health in general.

## Conclusion

The aim of this study was to gain insight into the opinions of the general public regarding the deservingness of health care and in particular insight into the role of level variation of allocation criteria on deservingness. We conclude that the general public finds medical need to be the most important criterion of a claimant to be considered deserving for collectively financed health care. However, people trade-off between all attributes, and different respondents—based on demography and ideology—do so differently. Thus, claimants with a medical condition are not considered unreservedly deserving; they are also held individually responsible to some extent, by means of their financial capacity, lifestyle, cooperation, and/or insurance choices. These results feed the debate on reforming health care allocation, in particular with regard to the balance between shared responsibility (solidarity) and individual responsibility.
